# The disappearing body: anorexia as a conflict of embodiment

**DOI:** 10.1007/s40519-021-01122-7

**Published:** 2021-03-05

**Authors:** Thomas Fuchs

**Affiliations:** Psychiatrische Universitätsklinik, Voss-Str. 4, 69115 Heidelberg, Germany

**Keywords:** Anorexia nervosa, Embodiment, Lived body, Object body, Body image

## Abstract

Anorexia nervosa is often regarded primarily as a disorder of the body image, with affected individuals submitting themselves to the dictate of a predominant model of slenderness. However, even though this frequently functions as a gateway to the disorder, the paper intends to show that the actual conflict in anorexia consists in a fundamental alienation of the self from the body. In order to analyze this alienation from a phenomenological point of view, the paper introduces the polarity of lived body (body-as-subject) and physical body (body-as-object). It then explores the phenomenology of anorexia, drawing on characteristic self-reports as well as on the phenomenological, psychoanalytic and cultural science literature. The anorexic conflict of embodiment arises in adolescence, where the body becomes an object of the other’s gaze in a special way. Starting with an attempt to comply with the ideal body image, the anorexic patient increasingly fights against her dependency on her body and its uncontrollable nature, above all its hunger and femininity. To be in total control of her body and to gain independence from it, becomes the source of a narcissistic triumph. Thus, in striving for autonomy and perfection, the anorexic patient alienates herself from her embodiment. This results in a radical dualism of ‘mind’ and ‘body’: pursuing the ideal of an asexual, angelic, even disappearing body. Anorexia is thus conceived as a fundamental conflict of embodiment.

## Introduction

Anorexia nervosa is often regarded primarily as a disorder of the body image, with affected individuals submitting themselves to the dictate of a predominant Western model of slenderness. According to the DSM 5, the disorder is characterized by a restriction of food intake and significant weight loss, “fear of becoming fat or gaining weight”, and a “distorted view of themselves and of their condition” ([1], 338f.). Dysfunctional beliefs and faulty cognitions about one’s body and its shape are usually taken to be the main causes of the disorder. However, this characterization overlooks the profound changes in bodily self-awareness in anorexic patients, which are expressed in the following typical statements:

It was as if I had to punish my body. I hated and detested it. If I let it be normal for a few days, then I would have to deprive it again. I feel caught in my body—as long as I keep it under rigid control, it can’t betray me. ([34], 278).

My body is not me. Again and again I take refuge in sport to feel it at all. When it is exhausted, when it hurts, then I feel it best. ([41], 71, transl. T. F.)

Another anorexic patient, after a binge eating attack, reports:

I was disgusted with myself, with my stuffed body. I felt like a sex offender at the mercy of his instinct to rape someone. (…) The taste of rotten eggs rose in me. I imagined how everything in me must have gone into a process of decay. ([28], 49, transl. T. F.).

Such descriptions already make it obvious that anorexia is more than a body image disorder – it is a fundamental disturbance of embodied self-experience. Legrand and Briend ([39], 56) rightly point to the radical difference “… between a woman who pursues a diet as she seeks to embody the images of beauty which are valued in her surroundings, and an anorexic subject who transforms her body so radically that she puts herself to death.” Even though striving for an ideal body image frequently functions as a gateway to the disorder, the actual conflict in anorexia consists in a severe alienation of the self from the body, which is increasingly experienced as an external, alien object and subjected to an authoritarian regime.

From a phenomenological point of view, the precondition for this alienation is an ambiguity of human embodiment itself, which to a certain extent prefigures the anorexic conflict. “The human being is his body, and at the same time, in reflecting upon his body, he stands outside it”, as Jaspers ([33], 354) put it. As we will see, this ambiguity is intensified in anorexia into a fundamental conflict between self and body, but also between demarcation and opening, autonomy and dependence, which is played out particularly at the borders and openings of the body and its nourishment. To analyze these conflicts, we must first describe the basic polarity of embodiment, namely between the subjective and objective body, between one’s pre-reflective and reflective relationship to the body, and finally between being-for-oneself and being-for-others. This will enable us, in the second part of the article, to better understand anorexia as a fundamental disorder of the embodied self.

### (1) Subject and object body

A common distinction in phenomenology is between the body as experienced and lived “from within”, i.e. the *subject-body (Leib)*, and the body as seen, observed or investigated from without, i.e. the physical or *object-body (Körper)*. At the most basic level, the lived or subject body conveys a vague interoceptive background feeling, for example of well-being or discomfort, of vitality, energy, or fatigue; it is also the source of drives, impulses and desires which the subject experiences either pre-reflectively or explicitly. All this is connected to a basic, pre-reflective self-affection or a sense of mineness—the *feeling of being alive* as the basis of conscious self-awareness ([24], 72f.). Further, the lived body also functions as a felt “resonance body” for all emotions that resound in it through various sensations and movement tendencies [25]. Finally, it tacitly mediates the subject’s sensorimotor engagement with the environment. In sum, the body is the general medium of our “being-towards-the-world” [44].

This lived body turns into a physical, objective body particularly when we become aware of it in a disturbing way, e.g. in fatigue, clumsiness, injury or illness. Having been a living bodily being before, I now realize that I have a physical (impeding, heavy, clumsy, vulnerable, even finite) body. In this way, the body *that I am* becomes an object *that I have*, and on which I am dependent—or rather, the body is *both* at the same time [22]. Thus, it has a double or ambiguous experiential status: an ongoing oscillation between these two bodily modes constitutes a fluid and hardly noticed foundation of all experiencing. The philosopher Helmuth Plessner [47] coined the term “excentric position” to characterize the ambiguous status of the human person being both inside her body, in the center of her world, and being outside of it, in reflective distance from pure centrality. Here a possible conflict can already be seen in the embodiment of the subject.

Importantly, the objectifying or external view of the body is closely linked to intersubjectivity. Sartre emphasized that my body becomes conscious to me in particular from another vantage point, namely when it is looked at by another person; it then becomes a ‘body-for-others’ ([48], 339). Through the other’s gaze, my body is turned inside out, as it were: it is no longer “… given merely as that which is purely and simply lived, rather this ‘lived experience’ becomes […] extended outside in a dimension of flight which escapes me. My body’s depth of being is for me this perpetual ‘outside’ of my most intimate ‘inside’ (ibid., 352). The mirror image reveals the ambiguity of realizing one’s body to be an object visible from the outside or for others and nevertheless *being* this object oneself.^1^ This ambiguity may be expressed as a tension between one’s primary embodied or 1^st^ person perspective and an internalized 3rd person perspective on my body and myself. According to Sartre, this tension also becomes an interpersonal conflict: while realizing that I am objectified or even reified by the other’s gaze, I have to withstand this gaze and (re-)affirm my own subjectivity, even if it is by objectifying the other in turn.

This intersubjective aspect of embodiment is of particular importance for understanding anorexia. If one’s own selfhood is to a certain extent reified by the other’s gaze and reduced to the external appearance of one’s body, then this is not just a matter of the body image. Rather, it may threaten one’s autonomy and even identity: I am no longer in control of my body, of my appearance, and to this extent, I am no longer in control of myself. Let us now examine how the possible conflicts within embodiment come to the fore in anorexia.

### (2) Anorexia as a disorder of embodiment

#### (a) Onset and release

Anorexia is mainly an illness of adolescence: the peak onset is between 15 and 19 years old, with about 90–92% of patients being female ([29], 43).^2^ Most girls develop anorexia after their bodies have begun to take on a more female shape and after their first menstruation. Importantly, puberty as such already intensifies the ambiguity and conflictual nature of human embodiment in several ways:

- Feminization, breast development and menarche entail new and unfamiliar forms of embodiment. The body takes on fuller, softer forms; its faster growth and change in appearance can lead to feelings of insecurity or alienation.
- The changes of the body’s shape are accompanied by alterations of the vital feelings; emerging drives and desires mean a *sexualization* of the body which can be experienced as promising, but also as irritating or even threatening: the lived body gains a life of its own, so to speak.
- At the same time, these changes lead to the body being exposed to the gazes of others in a new way. Only now does it become a ‘body-for-others’ in the full sense of the word, an object of evaluation, comparison and judgment. Teenage girls also increasingly attempt to model their body through training, diets, make-up, jewelry, piercings, etc. Thus, adolescence is the time when the *body image* acquires its true meaning, leading to constant self-evaluation and corresponding feelings of pride, embarrassment, shame or insufficiency. Reconciling one’s embodied sense of self with one’s bodily appearance becomes a crucial task, which already carries the germ of psychological derailments in itself.
- Finally, all these changes of puberty mean a central existential transition, namely the *departure from childhood*, which can be experienced with feelings of loss, sadness, abandonment and loneliness. One must first find oneself anew in the world. With this, the question of one's own *identity* often arises in a disturbing way, and feelings of inauthenticity and self-alienation may emerge: Am I really the person I think I am? Or am I just the person that others see in me?

These typical changes and conflicts of adolescence are, as we will now see, also crucial for the pathogenesis of anorexia. Usually, it starts with dissatisfaction with one's own body image and a resulting diet, which is then reinforced by one’s success and by the attention and recognition that the affected persons gain through their weight loss. The social objectification and resulting alienation of the body described by Sartre may thus be regarded as the major trigger of anorexia, as it has been emphasized by various authors [13, 38, 53, 54]. Undoubtedly this is favored by prevailing ideals of beauty and the ‘marketing’ of the body. Exposure to objectifying experiences and images may cause young girls or women to internalize others’ gazes, and from a feminist perspective, to accept the imposition on their body of a cultural ideal of thinness.^3^ They lose weight in order not to feel ‘too fat’ and to meet the ideal of the model on the catwalk, especially represented in the media.

However, this can hardly be a sufficient description nor explanation of the disorder. Certainly, cultural norms of beauty play an essential role in the onset of the condition. But its further course ceases to follow these norms. The gaze of others increasingly loses significance because it now becomes the anorexic’s own gaze, indeed also her own body feeling. She transforms the external evaluation into a regime of self-observation and self-assessment that detaches itself more and more from the usual norms of body image. The radicalization of starvation—even to the point of dying—apparently follows a different dynamic, which can no longer be explained by the mere pursuit of an ideal of slenderness and beauty. What does this dynamic consist of?

#### (b) Radicalization and reversal

Obviously, in anorexia the ideal of beauty and attractiveness driven to the extreme is reversed: the reification through the gaze of others turns into a self-reification, a deliberate emaciation of one’s own body, which precisely *eludes* the competition, the ‘market of beautiful bodies’. Paradoxically, the anorexic raises her demand for female slimness to an extreme where her body, in a sense, ceases to be that of a woman [57]—her female forms disappear, menstruation stops, libido and desire are lost, as well as any interest in the opposite sex. Reports from many patients indicate that this loss is actually highly welcome because her female body only triggers aversions or fear in them:

At first, the menstruation stopped, but I was very happy about that. Finally I could take revenge on it! It had given me a huge fright when it first appeared. How I had hated the thought: Now you are a physically mature woman, ready to sleep with a boy. ([28], 16, transl. T. F.).

I get scared when I discover the feminine side of myself. Fear of a female body. Fear of the onset of my menstruation. Fear of soft skin. Fear of becoming an adult. ([41], 93).

In the foreground, the anorexic pursues the ideal of thinness; however, her ‘hidden agenda’ is actually another, namely the refusal of feminization and adulthood.^4^ What is at stake for her, is ultimately to stop the maturation process, thus preventing the loss of the supposedly ‘asexual’ childhood. The radicalism with which she drives her emaciation forward points to a deeper insecurity, namely about her own lived body and identity in general. It is about the subjugation of the instinctive, libidinous, carnal body with its uncontrollable desire, which manifests itself in hunger as well as in sexuality. Obviously, the anorexic does not find a convincing female role model that could give her development a goal and make the future seem attractive. This of course points not least to a problematic relationship with her parents and their role models—a relationship which is usually characterized by a high degree of ambivalence [12, 36, 46].^5^

The reversal of the competition of bodies-for-others into the denial of the female role already indicates that the anorexic fight against nutrition is ultimately a desperate struggle for *autonomy and identity*. Most anorexic patients suffer from low self-esteem, accompanied by intense feelings of shame and latent or manifest depression [31, 58, 61]. They often feel that they have their lives determined by others, i.e. by social expectations and norms, such as the following patients:

I am a jumping jack who only moves when you pull his strings. I have nothing of my own, individual, distinctive; I am empty and do not know how what and who I am. ([27], 89, transl. T. F.)

I am a composite mosaic of pictures of other people. (ibid., 90).

By successfully controlling, monitoring and modelling their body, the patients thus try to compensate for their profound sense of lacking self-esteem, autonomy and identity. The anorexic can only experience her identity in the denial, in the radical otherness that makes her independent of all supplies of food and love: “I don’t feel hungry, I have no desire”—this means: I am self-sufficient and no longer need anything from outside [15]. Hence, the often fierce arguments with parents or relatives about refusing food, the arduous battles over more or less calories, are not only an expression of lack of insight into the illness: letting food into one’s own body is also the venue for the struggle for autonomy. I will come back to this later.

In sum, the anorexic gives a radical, even counterphobic answer to the dilemma of adolescence, namely the conflict between the bodily self and the body-for-others. The attractive or erotic female body is not an option for her, on the contrary: unable to withstand the objectifying gaze of the other and to affirm her own self against it, she evades the evaluating or desiring gaze, as it were, by fleeing forward. She does not find a balance between the body-for-others and the lived body; instead, she makes her body the object of self-mortification and thus of desexualization. For her, the body is not the living and unfolding realm of possibility, open to encounter and eroticism, but appears only as an alienated object of control, indeed increasingly as a hated adversary. Let us look at this alienation of the body a little closer.

#### (c) The alienated body

When the starvation of the body has become independent from external norms, the patients will realize that their initial desire to lose weight has turned into a *compulsion* to do so. The anorexic is literally obsessed with the subject of food and weight, while interest in almost everything else in life is lost [14]. In her hyper-reflexive attitude, the body is constantly at the centre, yet no longer as the *body-subject* but as a *body-object* from which she becomes more and more alienated. This often starts with the alienation of the mirror image, as analyzed in detail by Bowden [10]:

I looked in the mirror and suddenly didn’t quite know who that person was, couldn’t quite make a connection between her and me. ([30], 254).

What I perceived in the mirror image did not shock me like it seemed to shock other people... I just observed it, almost like I would do with an animal in a zoo. I saw its movements and bony bits, and I didn’t really relate them to any particular part of what I termed ‘me.’ ([11], 119).

The alienation of the body image can be interpreted as an increase in self-objectification, which already consists in the adoption of the other’s evaluative view on oneself. The body as mirrored by others and the body felt by oneself become increasingly detached and out of proportion [55, 56]. However, in the further course of the illness, the alienation begins to seize the lived and felt body too. As Legrand ([38], 733) notes, “eating disorders are not only characterized by over-objectification but also by weakened body-ownership”. Patients no longer feel at home in their bodies, they report feelings of numbness, dullness or rigidity, which transform the body almost into an inanimate object. In a larger study, using an embodiment-related questionnaire, Stanghellini et al. [53]found a feeling of being “extraneous from one’s body” to be present in the majority of patients.^6^

This alienation is associated with a loss of emotional intensity, which is normally communicated through the resonance of the lived body [25]. Alexithymia, i.e. difficulties in identifying and describing one’s emotions, is commonly associated with eating disorders [3, 35], it also manifests itself in an over-intellectual, rationalistic style of communication [16]. The patients often experience their starvation as a “self-anaesthetization” [37], a numbness which frees them from negative feelings, tension, worries and depression, and which may be regarded as a dysfunctional form of affect regulation [26]. They may describe it as retreating into a numb and protective “shell”, as if there was a “screen” between themselves and reality [17]:

… it’s almost like everything became a bit of a blur and like I was living in this kind of cloud where I didn’t really feel anything at all because I was so nutritionally deprived. ([37], 460).

The process of self-objectification thus leads to a growing detachment of the anorexic person from her body, reducing it to its physical dimension—its measurable size, weight, caloric intake, etc.—and keeping it under constant scrutiny. Rituals of measuring, weighing, calorie counting, food selection and intake, training and exertion are increasingly restricting her life [53]. More and more she experiences her body as a distorted object: as bulky, massy, stuffed, still ‘too fat’ and ‘too heavy’. This may be described as a ‘corporealization’ of the lived body, a phenomenon that is also characteristic of acute shame and depression [22, 23]: instead of tacitly mediating the subject’s relations to the world, the body comes to the fore as an insistent, obstinate, unruly object. Moreover, for the anorexic it becomes an instrument of self-discipline that is mercilessly starved or even an enemy whose impulses must be controlled and fought. Of course, this ultimately manifests the latent contempt and hate that patients feel towards themselves.

The anorexic thus fights against the female, soft, swelling body shapes that develop during puberty, trying to get rid of her feeling of a stuffed and amorphous body—“a structureless, misshapen, gelatinous, meaningless mass”, as a patient described it ([27], 79). Against this, she places the lean, pointed, angular body with its hard, protruding bones:

One day I will be thin enough. Just the bones, no disfiguring flesh, just the pure, clear shape of me. Bones. ([51], 9).

I want to avoid curves—I always avoided looking like a woman … I do not want to have the kind of body females have. ([12], 120).

My body is hard, angular and compact. There is nothing soft, round, and it should remain that way for all eternity, Amen. ([20], 173, transl. T. F.)

It becomes clear that anorexia also serves the purpose of combating the threatening permeability and dissolution of the solid body, which manifests itself particularly in digestion, but also in bodily fluids such as menstruation. For the anorexic, this liquefaction means decay and triggers feelings of disgust:

So there’s nothing to do but lie here and feel my body bloat and rot, rot and spread, spread and deliquesce, decompose. […] Because there’s all this putrefaction, fermentation, and resulting acid, what actually is in the stomach is a mass of spoiled, rotting, foul-smelling food. ([51], 152).

Disgust is also the affect of *expulsion*, which is directed against food as a foreign body. For a patient interviewed by Warin [60], food seemed to contaminate her body: once inside the mouth, it had crossed from “the external into the internal world … it’s inside you, it has been imbibed, and internalized and there is a sense of feeling dirty, and impregnated with something …” ([60], 81).

Nutrition thus stands for all kinds of intrusion of the foreign that could violate one’s physical boundaries and destroy one’s autonomy. Metabolism means exchange with the environment, but also dependence on it; starvation and vomiting, on the other hand, are equivalent to separation, purification and expulsion. Seen from an intersubjective point of view—and the lived body is indeed always an intersubjective body—this would also be a way to express that: “I have sharp contours, I’m not soft or open, I do not merge with you; you cannot penetrate into me.” Thus, via her body, the anorexic patient struggles relentlessly for her autonomy.

#### (d) The disappearing body

The result of this struggle is a radical dualism of self and body: on the one hand, the anorexic patient exercises a quasi-military regime over her body; she closes it off from exchange with the environment and suppresses its libidinal needs. She constructs her body as a self-contained, closed system whose boundaries “may then be crossed only on her authority, and under extremely controlled and ritualized circumstances” ([40], 487). On the other hand, her aim is *to negate the heaviness and materiality of the body* in general. What she pursues is not only thinness but also lightness, weightlessness, or in one word: *disembodiment*, expressed in the ultimate fantasy of floating in the air.

I feel high, like I am separated from my body. I love that feeling—it is like being a spirit. [4]

Physiological consequences of constant starvation support this disembodiment: high levels of glucocorticoid and serotonin can make hunger feel euphoric and turn it into something addictive [7]. States of exhilarating lightness, floating, and a heightened transparency of reality seem to realize the ideal of purity, clarity and dematerialization that the anorexic pursues. Autarky—to be in total control of her body, to gain independence from it, from food as well as from others—combines with a feeling of moral superiority and becomes the source of a grandiose triumph, a “self-destructive omnipotence” ([19], 25):

I could control myself! I could do without! I was strong! I really enjoyed watching the others eat and seeing how it made them weaker and weaker in my eyes—those who were otherwise so strong. Yes, not only did they become weaker, they were suddenly worse people than me. Unlike me, they needed something as primitive as food. They were not in control of themselves, they lacked control. They were greedy, they were like wild, run-down animals that pounced on something to eat. Not eating was my strength alone, and no one could take it away from me. ([27], 62, transl. T. F.).

I wanted to belong to the elite with my anorexia and associated it with being something extraordinary, special, extravagant, unique. I wanted to demonstrate that I was a spiritual person, a person to whom material greed and passion were alien (ibid., 105).

What is opposed to the alienated body, then, is the pure mind, soul, will, or spirit—a principle of purity and transparency which seems to be imprisoned in the body ([9], 139f.). Thus, in anorexia the basic tension of being a body and having a body turns into a dichotomy that is reminiscent of the Platonic and Gnostic ideas of the body as the “dungeon of the soul”:

I feel caught in my body—as long as I keep it under rigid control, it can’t betray me. ([34], 278).

The historical connection between religious asceticism and voluntary starvation by women in Western Europe during the medieval and early modern periods has often been emphasized in the context of anorexia [2, 5, 6]. Fasting and refusing food had a religious meaning and were associated with sexual renunciation and virginity throughout this historical period. In this respect, the psychiatric “discovery” of anorexia towards the end of the nineteenth century can also be seen as the “medicalization” of a behavior that until then belonged to a religious context [59]. Despite the secularization that has taken place since then, the similarly ascetic motivation is still unmistakable and points to a kindred, rejecting attitude toward the libidinal, carnal body. In her desperate pursuit of perfection, today’s anorexic, too, is alienating herself from her earthly, bodily being. Her ideal is the non-physical, asexual, angelic body. Her actual role model is not the model on the catwalk but the saint (even if she would deny this at the conscious level). Indeed her ultimate desire is to make the material, dirty, hungry and covetous body dissolve and *disappear*.

I wish I could get out of my body entirely and fly. ([42], 141).

Please dear God, help me. I want to get out of my body, I want to get out! ([62], 200).

Similarly, Binswanger’s Ellen West saw a crucial meaning of her self-starvation in the “ideal of being too thin, of *being without a body*” ([8], 251, emphasis added). This dualistic ideal marks the extreme of the rupture that can arise in the relationship between humans and their body. Ultimately, it is their excentric position that makes this split possible. But it also becomes clear that the anorexic has maneuvered herself into an irresolvable contradiction: namely between the longed-for dissolution of her body on the one hand, and its reification and ‘corporealization’ on the other. Instead of becoming one with her lived body (and thus being able to feel the lightness of ‘flow’-experiences, for example), she is all the more chained to her reified, material body: she cannot get rid of it, and all her thinking and doing only revolves around it.

#### (e) The intersubjective body

We have seen how the body of the anorexic becomes the site of a relentless struggle for demarcation, autonomy and identity. But it would be too one-sided to understand the refusal to eat only as the refusal to relate to the world and to others. The ambivalence of the body concerns not only the subjective and objective body; it also implies the contradiction between dependence and independence, of being open and self-contained at the same time. The body is thus the arena most suitable for the patients to express this conflict and exert control over either dimension:

With my anorexia, I could have the guarantee that they (my parents) would continue to care for me, but I could also prove independence and rebellion. I held the reins, I was the absolute center of my family. ([27], 114, transl. T. F.).

Moreover, the body also means nourishing and being nourished, as an intersubjective experience present from early childhood on. Eating has the meaning of preparing food together, of the common meal, and of the conversations associated with it. All this points not to the Sartrean aspect of the objectified body-for-others or the body image, but to the dimension of *intercorporeality* [45], or to the *intersubjective body* [38].

What then is the intersubjective meaning of the anorexic body?—On the one hand, anorexic patients frequently refuse to eat with their families, never eat in public and withdraw from social engagements, even more from intimate contacts. On the other hand, it is precisely because of their anorexia that they attract all the attention and become the center of the family. Their body sends out an unmistakable appeal; everybody can see its deplorable and even alarming state. Hunger calls for the response of others, especially parents, for to see their own child starve or even starve to death is utterly unbearable.

Hence, “the anorexic body is rich in semiotic sense” ([52], 172), but this sense is ambiguous: it signals a desperate striving for self-worth, autonomy and demarcation, but at the same time an equally great striving for recognition, protection and love. The latter is admittedly repressed and only recognizable by the pitiful appearance of the girl, who nevertheless haughtily and aggressively rejects all attempts to help. What she wants more than anything else is to be acknowledged and taken care of, but she can only express this wish with her body.

You think you are worthwhile only if you do something very special, something so great and dazzling that your parents and other people you care about will be impressed and admire you for being super-special. ([12], 137f.)

With my anorexia I wanted to take revenge on my parents. I wanted to provoke them, to draw them out of their reserve and insensitivity. ([27], 114, transl. T. F.).

The anorexic body is by no means just the objectified body; it is also a visibly vulnerable, fragile and thus thoroughly expressive body. It communicates what the patient is otherwise unable to communicate: “The anorexic subject is thus hungry for the other, not for who the other is as such, but for his capacity to respond to her, thereby confirming that he recognizes her as a subject who is calling with her demand” ([39], 60). It is not without reason that the contradictory signals of the anorexic body have also been interpreted as a paradoxical attempt to *increase* the attachment to the family and family cohesion. From a family systems approach, the patient’s behavior is considered to aim at and contribute to the preservation of family bonds and of her belongingness, which she sees as threatened [46, 49, 50]. The body of the anorexic becomes the center of the family, around which all fears, conflicts, appeals and wishes revolve. Yet the more the symptom of starvation participates in the homeostatic balance of the family, the less it can be abandoned.^7^

In sum, eating disorders are deeply relational: the feeding or starving, devouring or fasting, overweight or underweight body is always also the body-for-others; not in the Sartrean sense, however, but as a body that expresses a relationship, communicates a message for others, makes a claim and an appeal to them. While the rigid, bony body imperiously rejects any touch, embrace or even fusion, it nonetheless longs for bodily closeness and protection. In this sense, anorexia may ultimately be regarded as a disorder of intercorporeality as well.

## Conclusion

I have interpreted anorexia as the expression of a fundamental conflict within corporeality, namely as an intensification of the dialectic of subject- and object-body. It is no coincidence that the onset of the disorder lies in puberty when the conflict between the primary, spontaneous bodiliness and the reflected body-object perceived from outside actually unfolds.

The disturbance usually begins with the realization of the reifying gaze of the other and one’s own body image, which the anorexic first tries to adjust to the prevailing ideal. But the radicalism with which this ideal is pursued points to an underlying deeper insecurity, namely towards one's own embodiment and femininity in general. Ultimately, the anorexic is concerned with the subjugation of the libidinal body, the refusal of feminization and the defiance of childhood’s end. In anorexia’s further course, the perceived reification by others turns into self-reification, a starvation of one’s own body, which completely eludes the competition of beautiful bodies. On a deeper level, this means the attempt to arrest the course of biological life which would lead to maturation, sexuality, and adulthood. The patient thus experiences an *inhibition of becoming*; her life is “detained in a sort of adolescent or preadolescent world” ([16]: 278).

As a result, the anorexic feels increasingly alienated from her own body, which becomes the devalued object of her domination, even the hated enemy of her absolute self-will: everything soft, swelling, dissolving is supposed to transform into hardness and firmness. The disgust towards food, especially fat, serves to repel or expel the amorphous and shapeless, from which the anorexic feels threatened. At the same time, she experiences the grandiose triumph of controlling her body, of no longer being dependent on its drives and needs. Purity instead of mixture, spirituality instead of materiality, self-sufficiency and demarcation instead of dependence are the commandments of her fanatic asceticism. In her unyielding quest for perfection, the anorexic alienates herself from her earthly, bodily being. For her, the fundamental human tension of being a body and having a body becomes a radical dualism of mind and body, spirit and matter, and thus, a fight against herself, indeed a fight to the death.

Anorexic patients have sufficient will power to gain a substitute identity through the successful submission of their body and through the radical negation of all feminine role models. The triumph of independence and self-sufficiency, of course, can only be achieved at the price of an ever more cruel regime to which they subject themselves. The reverse side of this regime is the implicit, desperate call for recognition, protection and care, which can no longer be communicated other than through the expressiveness of the tortured body.

The solution to this fundamental conflict of embodiment ultimately lies in intercorporeality: the anorexic must learn to accept the natural development and maturation of her body, which can connect with other bodies; she must learn to accept the dependence and neediness, which belongs to the basic conditions of embodied existence: dependence on food, contact and relationship. In this way, she can also learn to experience without fear the pleasure, joy and fusion that the lived body can provide; and she can experience that it is precisely in the relationship, openness and intercorporeality with others that she can gain a genuine sense of identity without losing her autonomy. The basic conflict of human embodiment between lived and object body, autonomy and dependency, self and other, thus contains at the same time the outline of its solution.

## Footnotes

1 Looking at a mirror for a longer time may therefore cause a slight alienation, namely between my lived body as felt from the “inside” and my body image which faces me from “outside”.

2 Because of this ratio, the following description is largely restricted to female patients.

3 See Lester [40]. In his seminal study *The Absent Body*, Drew Leder also speaks of the “social dys-appearance” of the body, resulting from a split between the self and an alienating other ([63], 96). The lived body is turned into an object precisely because the primary empathic communication of bodies, Merleau-Ponty’s intercorporeality, is thwarted.

4 Already Janet [32] assumed that *Anorexia Mentalis* reflected the patient’s refusal to assume the female sexual role.

5 This cannot be examined in more detail in this context. My paper does not aim to provide a causal explanation of anorexia, which is certainly of a complex nature and would have to include biological, social, biographical and psychodynamic factors. It attempts instead a phenomenological analysis of the subjective experience of the condition and its essential, particularly bodily structures and dynamics.

6 Esposito and Stanghellini [18] interpret these findings as evidence that the disturbances of bodily self-experience are a primary deficit of anorexics (“hypo- and dis-coenaesthesia”), which results in their excessive orientation to the gaze of others as conveying a substitute sense of identity. However, this assumption of a primary disturbance of coenaesthetic experience does not seem to be proven by the merely cross-sectional findings. It also seems unlikely, since a separate genesis would have to be postulated for such a primary disturbance of coenesthesia or interoception. Finally, if at the origin of anorexia there is always “a gaze that wounds”, as the authors assume (ibid., 294), it is difficult to see how this gaze of the other could convey a substitute identity.

7 Recent research has pointed to the reinforcing effect of parents’ or relatives’ assisting behavior upon the patient’s symptoms [21].

## References

[1] APA (2013) Diagnostic and statistical manual of mental disorders, 5th ed. (DSM-V). American Psychiatric Association, Washington

[2] Banks CG (1996). “There is no fat in heaven”: religious asceticism and the meaning of anorexia nervosa. Ethos.

[3] Beadle JN, Paradiso S, Salerno A, McCormick LM (2013). Alexithymia, emotional empathy, and self-regulation in anorexia nervosa. Ann Clin Psychiatry.

[4] Beeken C (1997) My body, my enemy. My thirteen year battle with anorexia nervosa. Thorsons, London

[5] Bell R (1985). Holy anorexia.

[6] Bynum C (1987). Holy feast and holy fast. The religious significance of food to medieval women.

[7] Bergh C, Södersten P (1996). Anorexia nervosa, self–starvation and the reward of stress. Nat Med.

[8] Binswanger L (1958) The case of Ellen West. In: May R (ed) Existence.Simon & Schuster, New York

[9] Bordo S (1993). Unbearable weight: feminism, western culture and the body.

[10] Bowden H (2012). A phenomenological study of anorexia nervosa. Philos Psychiatry Psychol.

[11] Bowman G (2006). A shape of my own.

[12] Bruch H (1978). The golden cage. The enigma of anorexia nervosa.

[13] Castellini G, Trisolini F, Ricca V (2014). Psychopathology of eating disorders. J Psychopathol.

[14] Charland LC, Hope T, Stewart A, Tan J (2013). Anorexia nervosa as a passion. Philos Psychiatry Psychol.

[15] Dignon A, Beardsmore A, Spain S, Kuan A (2006). ‘Why I Won't Eat’: Patient testimony from 15 anorexics concerning the causes of their disorder. J Health Psychol.

[16] Doerr-Zegers O, Pelegrina-Cetran H (2020) Phenomenology of corporeality (and spatiality) in Anorexia Nervosa with a reference to the problem of its temporality. In: Tewes C, Stanghellini G (eds.) Time and body. Phenomenological and psychopathological approaches. Cambridge University Press, Cambridge, pp 163–281

[17] Eli K (2018). Striving for liminality: eating disorders and social suffering. Transcult Psychiatry.

[18] Esposito CM, Stanghellini G (2020). The pathogenic and therapeutic potential of the gaze of the other in the clinic of “eating disorders”. Psychopathology (Online First).

[19] Farrell E (1995). Lost for words: the psychoanalysis of anorexia and bulimia.

[20] Fechner A (2007). Hungrige Zeiten. Überleben mit Magersucht und Bulimie.

[21] Fox JR, Whittlesea A (2017). Accommodation of symptoms in anorexia nervosa: a qualitative study. Clin Psychol Psychother.

[22] Fuchs T (2002). The phenomenology of shame, guilt and the body in body dysmorphic disorder and depression. J Phenomenol Psychol.

[23] Fuchs T (2005). Corporealized and disembodied minds. A phenomenological view of the body in melancholia and schizophrenia. Philos Psychiatry Psychol.

[24] Fuchs T (2018). Ecology of the brain. The phenomenology and biology of the embodied mind.

[25] Fuchs T, Koch S (2014) Embodied affectivity: on moving and being moved. Front Psychol Psychol Clin Set 5: 508 10.3389/fpsyg.2014.00508 PMC4047516 24936191

[26] Gaete IM, Fuchs T (2016). From body image to emotional bodily experience in eating disorders. J Phenomenol Psychol.

[27] Gerlinghoff M, Backmund H, Mai N (1988). Magersucht. Auseinandersetzung mit einer Krankheit.

[28] Graf A (1985). Die suppenkasperin. Geschichte einer Magersucht.

[29] Hoek HW (2006). Incidence, prevalence and mortality of anorexia nervosa and other eating disorders. Curr Opin Psychiatry.

[30] Hornbacher M (1999). Wasted.

[31] Ivarsson T, Råstam M, Wentz E, Gillberg IC, Gillberg C (2000). Depressive disorders in teenage-onset anorexia nervosa: a controlled longitudinal, partly community-based study. Comp Psychiatry.

[32] Janet P (1903). Les obsessions et la psychasthénie.

[33] Jaspers K (1997) General Psychopathology. Trans J Hoenig M. W. Hamilton. Johns Hopkins University Press, Baltimore

[34] Kaplan LJ (1984). Adolescence—the farewell to childhood.

[35] Kessler H, Schwarze M, Filipic S, Traue HC, von Wietersheim J (2006). Alexithymia and facial emotion recognition in patients with eating disorders. Int J Eat Disord.

[36] Kluck AS (2008). Family factors in the development of disordered eating: Integrating dynamic and behavioral explanations. Eat Behav.

[37] Lavis A (2018). Not eating or tasting other ways to live: a qualitative analysis of ‘living through’and desiring to maintain anorexia. Transcult Psychiatry.

[38] Legrand D (2010). Subjective and physical dimensions of bodily self-consciousness, and their dis-integration in anorexia nervosa. Neuropsychologia.

[39] Legrand D, Briend F (2015). Anorexia and bodily intersubjectivity. Eur Psychol.

[40] Lester RJ (1997). The (dis)embodied self in anorexia nervosa. Soc Sci Med.

[41] Lena S (2006). Auf Stelzen gehen. Geschichte einer Magersucht.

[42] Liu A (1979). Solitaire.

[43] Lucas AR, Beard CM, O’Fallon WM, Kurland LT (1991). 50-year trends in the incidence of anorexia nervosa in Rochester, Minn.: a population-based study. Am J Psychiatry.

[44] Merleau-Ponty M (1958) Phenomenology of Perception. Transl. by C Smith. Routledge & Kegan Paul, London

[45] Merleau-Ponty M (1960) Le philosophe et son ombre. Signes*.* Éditions Gallimard, Paris

[46] Minuchin S, Rosman BL, Baker L (1978) Psychosomatic families: anorexia nervosa in context. Harvard University Press, Cambridge

[47] Plessner H (2019) Levels of organic life and the human: An introduction to philosophical anthropology (original publication 1928). Transl. M. Hyatt, P. Honenberger. Fordham University Press, New York

[48] Sartre J-P (1969) Being and Nothingness. Translated by Hazel E. Barnes. Routledge, London

[49] Selvini Palazzoli M (1974). Self starvation.

[50] Selvini Palazzoli M, Boscolo L, Cecchin G-F, Prata G (1974). The treatment of children through brief therapy of their parents. Fam Process.

[51] Shute J (1992). Life-size.

[52] Skårderud F (2007). Eating one's words, Part I: ‘concretised metaphors’ and reflective function in anorexia nervosa—an interview study. Eur Eat Disord Rev.

[53] Stanghellini G, Castellini G, Brogna P, Faravelli C, Ricca V (2012). Identity and eating disorders (IDEA): a questionnaire evaluating identity and embodiment in eating disorder patients. Psychopathol.

[54] Stanghellini G (2019). Embodiment and the other's look in feeding and eating disorders. World Psychiatry.

[55] Stanghellini G (2019). The optical-coenaesthetic disproportion in feeding and eating disorders. Eur Psychiatry.

[56] Stanghellini G, Ballerini M, Mancini M (2020). The optical-coenaesthetic disproportion hypothesis of feeding and eating disorders in the light of neuroscience. Front Psychiatry.

[57] Svenaeus F (2013). Anorexia nervosa and the body uncanny: a phenomenological approach. Philos Psychiatry Psychol.

[58] Troop NA, Allan S, Serpell L, Treasure JL (2008). Shame in women with a history of eating disorders. Eur Eat Disord Rev.

[59] Vandereycken W, Van Deth R (1994). From fasting saints to anorexic girls: the history of self-starvation.

[60] Warin M (2003). Miasmatic calories and saturating fats: fear of contamination in anorexia. Cult Med Psychiatry.

[61] Wilksch S, Wade TD (2004). Differences between women with anorexia nervosa and restrained eaters on shape and weight concerns, self-esteem, and depression. Int J Eat Disord.

[62] Woods J (1981) I was starving myself to death. Mademoiselle 87(5)

[63] Leder D (1990) The absent body. University of Chicago Press, Chicago

